# Endovascular Repair for Abdominal Aortic Rupture Caused by Periaortic Mantle Cell Lymphoma

**DOI:** 10.3400/avd.cr.20-00105

**Published:** 2020-12-25

**Authors:** Yukihiro Matsuno, Shohei Mitta, Yukio Umeda, Ryota Watanabe, Yoshio Mori

**Affiliations:** 1 Department of Cardiovascular Surgery, Gifu Prefectural General Medical Center, Gifu, Gifu, Japan; 2 Department of Cardiology, Gifu Prefectural General Medical Center, Gifu, Gifu, Japan

**Keywords:** endovascular repair, aortic rupture, lymphoma

## Abstract

A 72-year-old man was referred to our hospital for the suspicion of ruptured abdominal aortic aneurysm. Before admission, he was suspected of having a malignant lymphoma and underwent excisional biopsy in his right groin. A contrast enhanced computed tomography scan revealed a massive retroperitoneal hematoma with an extravasation arising from the infrarenal abdominal aorta coexisting with an extensive retroperitoneal mass surrounding the aorta. An emergency endovascular aneurysm repair was performed and the postoperative course was uneventful. After the treatment, histological examination of the previous biopsy confirmed the diagnosis of mantle cell lymphoma.

## Introduction

Aortic rupture is a dangerous condition associated with several etiologies including aortic aneurysm, aortic dissection, penetrating atherosclerotic ulcer, infection, and trauma. This condition, which is due to secondary invasion of neoplasm, is a rare clinical feature. Herein, we present a case of endovascular repair for infrarenal abdominal aortic rupture caused due to invasion by periaortic mantle cell lymphoma.

## Case Report

**Figure 1:**
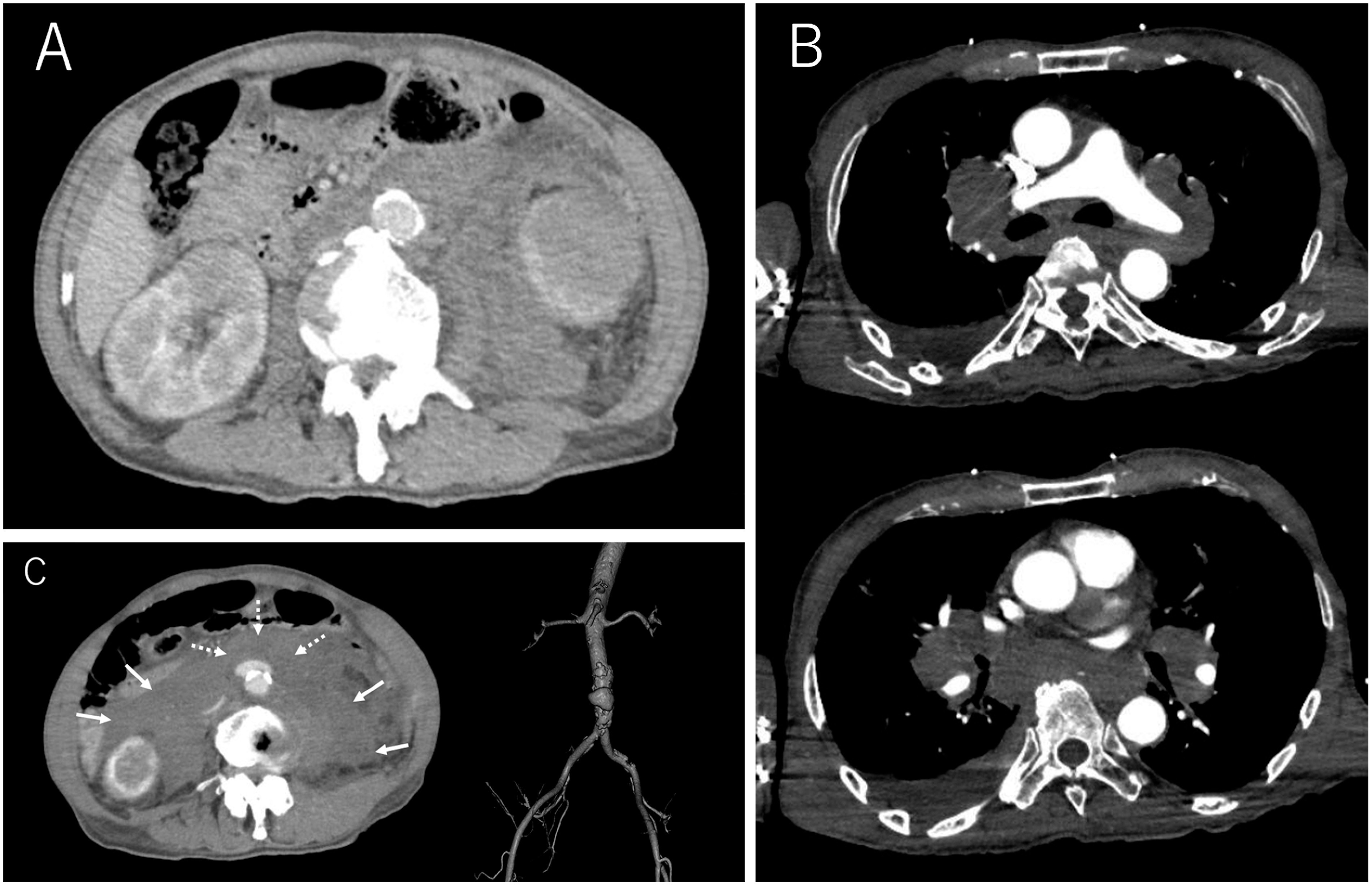
Fig. 1 Preoperative contrast enhanced computed tomography (CT) scan.

A 72-year-old man was admitted to a local hospital with complaints of general fatigue and body weight loss. A computed tomography (CT) scan of his chest was performed and showed lymphadenopathy in his whole body, including axillar, hilar, mediastinal, and retroperitoneal regions (**Figs. 1A** and **1B**). The patient was suspected of having malignant lymphoma and underwent excisional biopsy in his right groin. One week after the biopsy, he suddenly presented with a complaint of acute abdominal pain. An emergency contrast enhanced CT scan was performed and revealed a massive retroperitoneal hematoma. Consequently, he was transferred to our hospital for the suspicion of ruptured abdominal aortic aneurysm.

(**A**) A CT before biopsy shows a retroperitoneal mass surrounding the abdominal aorta, which appears to be almost of normal caliber with slight expansion (axial view). (**B**) Lymphadenopathy in the hilar and mediastinal regions. (**C**) Massive retroperitoneal hematoma with active extravasation from the infrarenal abdominal aorta (solid arrow) coexisting with an extensive retroperitoneal mass surrounding the aorta (dotted arrow).

Upon arrival, he was drowsy, pale, and complained of progressively worsening abdominal pain. His blood pressure was 90/50 mmHg, the pulse rate was 110 beats/min, the hemoglobin was 10.0 g/dL, and the oxygen saturation was 99%. He had no past history of hypertension. A contrast enhanced CT scan was performed repeatedly and revealed a massive retroperitoneal hematoma with active extravasation arising from the infrarenal abdominal aorta coexisting with an extensive retroperitoneal mass surrounding the aorta (**Fig. 1C**). The aorta appeared to be almost of normal caliber, with slight expansion. Radiologic interpretations by a specialist pointed out the density difference between the hematoma and the periaortic soft tissue in the retroperitoneal lesion. As a result, we suspected abdominal aortic rupture caused by invasion by the malignant lymphoma and decided to perform an emergency operation. Considering the risk of tight adhesion between the aorta and the lymphoid tissues, conventional open repair could have been technically difficult. Therefore, we chose to perform endovascular aneurysm repair (EVAR) as an alternative.

**Figure 2:**
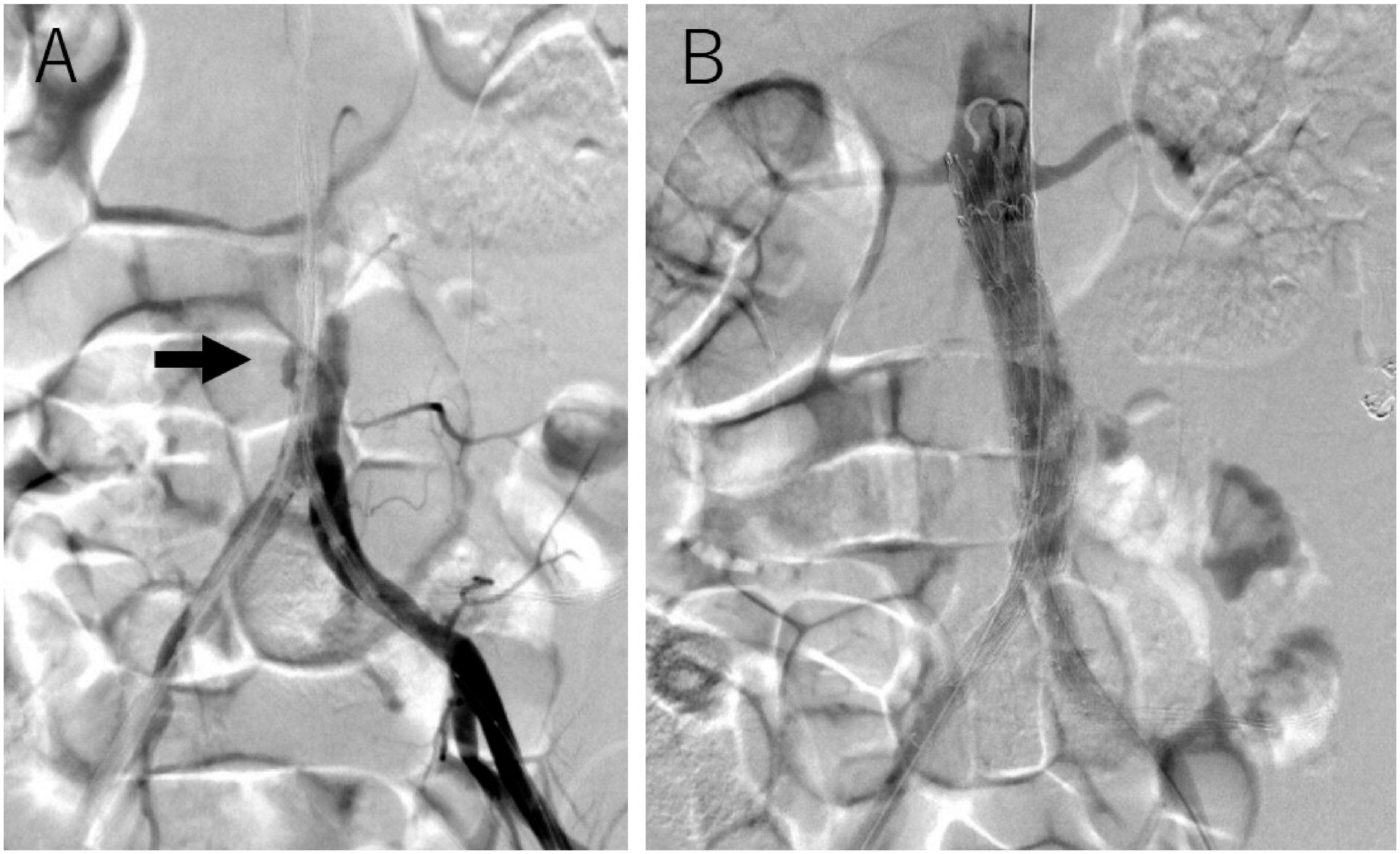
Fig. 2 Intraoperative angiography.

An emergency EVAR was performed under local anesthesia with mild sedation. Initial angiography confirmed an active extravasation arising from the infrarenal abdominal aorta (**Fig. 2A**). An AFX® endoprosthesis (Endologix, Irvine, CA, USA) was placed below the origin of the bilateral renal arteries covering the ruptured aortic segment using standard techniques. In order to avoid type IA endoleak, another extension cuff was placed in the proximal region. Completion angiography showed the endoprosthesis was in a good position and no evidence of endoleak was observed (**Fig. 2B**).

(**A**) Initial angiography showing active extravasation arising from the infrarenal abdominal aorta (arrow). (**B**) Post deployment angiography showing stent graft in situ, with exclusion of the rupture point.

**Figure 3:**
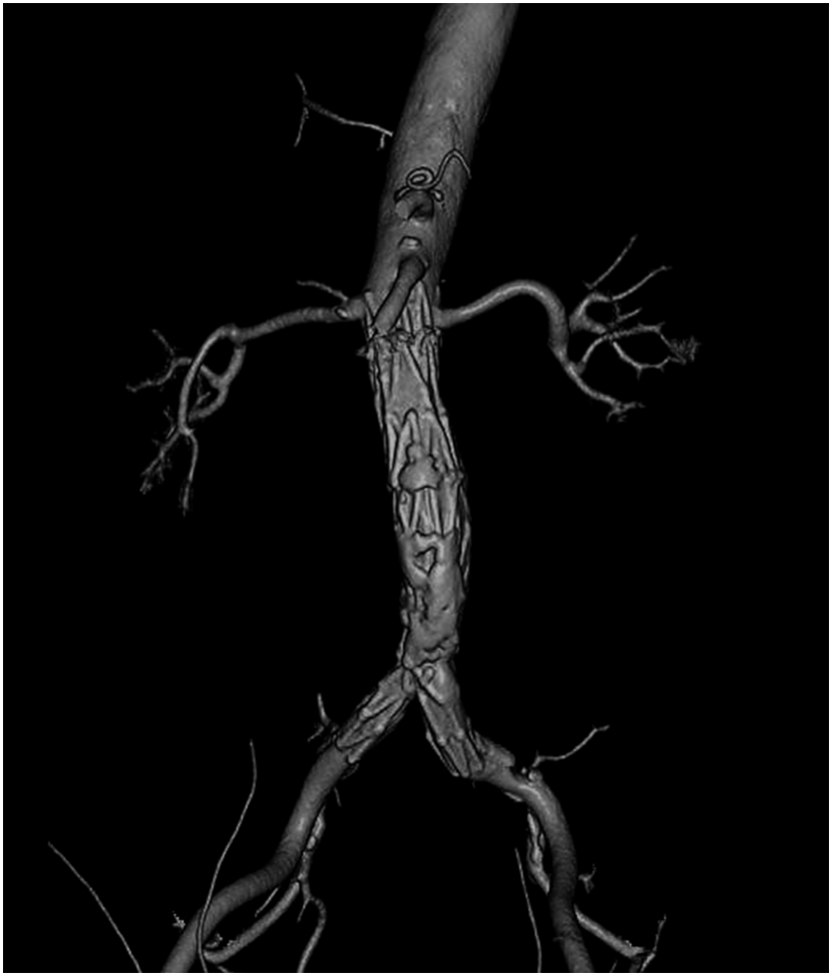
Fig. 3 Postoperative 3-dimensional computed tomography scan showing complete sealing the rupture point with no evidence of endoleak.

The patient’s postoperative course was uneventful and postoperative CT scan demonstrated complete sealing of the rupture point with no evidence of endoleak (**Fig. 3**). Five days after the treatment, histological examination of the previous biopsy confirmed the diagnosis of mantle cell lymphoma (MCL), which is one of several subtypes of B-cell non-Hodgkin lymphoma. Afterwards, the patient was transferred to a specialized hospital for the purpose of receiving chemotherapy for MCL.

## Discussion

Aortic rupture is an extremely dangerous and rare condition that occurs as a result of decrease in the strength of the aortic wall where the systemic pressure is more than the wall strength, or because of any external destruction or damage to the aortic wall. Aortic aneurysm is the most common cause of aortic rupture, and other causes include aortic dissection, intramural hematoma, penetrating atherosclerotic ulcer, infection, inflammation, or trauma.

Aortic rupture caused by secondary invasion of neoplasm is a rare clinical occurrence. Lymphoma is the most common type of blood neoplasm. It affects lymphocytes and is divided into the following two main categories: Hodgkin lymphoma and non-Hodgkin lymphoma.^1)^ MCL is a relatively uncommon subtype of non-Hodgkin lymphoma that is associated with poor prognosis due to aggressive clinical course, low sensitivity to traditionally used chemotherapy, and high relapse rates.^2)^ Several clinical cases of aortic aneurysms and ruptures, or aortic dissections due to secondary invasion by periaortic lymphoma have been previously reported.^3,4)^ According to those previous reports, underlying mechanisms of aneurysmal degeneration or rupture in patients with periaortic lymphoma were attributed to direct invasion or compression of the aortic wall by lymphoma tissues. That is, lymphomatous tissues can invade the normal aortic wall and weaken the structural integrity of the aorta resulting in subsequent rapid aneurysmal dilation or rupture. Furthermore, Sprague et al. suggested that inflammatory cytokines released by lymphocytes could play a role in vascular remodeling resulting in weakening of the structural integrity of the vascular wall.^5)^ In the present case, even though the abdominal aorta was of almost normal caliber with slight expansion, aortic rupture occurred. We suggest that the aortic rupture might be associated with complex probable causes as mentioned above, although we were unable to identify definitive histological evidence of lymphocytic cell invasion in the aortic wall.

It is difficult to distinguish between hematoma due to aortic rupture and periaortic lymphoid tissue because their appearances are similar on CT scan. In the present case, a radiologist pointed out the difference of the CT density between hematoma and periaortic soft tissue in the retroperitoneal lesion. Although there have been a few reports in which magnetic resonance imaging was used to confirm the diagnosis,^6)^ in the acute setting of aneurysm rupture, such a time-consuming inspection might not be practical.

Conventional open surgical repairs for aortic aneurysm or rupture due to secondary invasion of lymphoma have been previously reported by several authors.^7)^ However, tight adhesion between the aneurysm and lymphoid tissues have the potential to make emergency open repair technically difficult. On the other hand, EVAR enables safe and less-invasive treatment without laparotomy. Meta-analysis data indicate that the perioperative mortality after EVAR is lower than that after open repair for ruptured abdominal aortic aneurysm.^8)^ Furthermore, several studies have reported that EVAR is more advantageous than open repair in patients with synchronous intra-abdominal malignancy.^9)^ Considering these reports, endovascular repair provides an effective and alternative approach in a situation when conventional open repair may be hazardous or impossible.^10)^ We also chose EVAR considering the possibility of a tight adhesion between the aorta and lymphoid tissues; consequently, we had a good success.

## Conclusion

We presented a case of endovascular repair for infrarenal abdominal aortic rupture due to invasion of periaortic MCL. Endovascular repair can be considered as a safer and more attractive option when anatomically feasible in the acute setting of aortic rupture, especially in patients with several comorbidities. We recommend paying careful attention to aortic aneurysm or rupture due to invasion when encountering a sudden onset of abdominal or back pain in patients with lymphoma.

## References

[1] Satou A, Bennani NN, Feldman AL. Update on the classification of T-cell lymphomas, Hodgkin lymphomas, and histiocytic/dendritic cell neoplasms. Expert Rev Hematol 2019; 12: 833-43. 31365276 10.1080/17474086.2019.1647777 PMC6763378

[2] Jain P, Wang M. Mantle cell lymphoma: 2019 update on the diagnosis, pathogenesis, prognostication, and management. Am J Hematol 2019; 94: 710-25. 30963600 10.1002/ajh.25487

[3] Kreel L, Bydder G. Associated lymphoma and abdominal aortic aneurysm demonstrated by computed tomography. J Comput Tomogr 1980; 4: 209-14. 7261652 10.1016/0149-936x(80)90008-9

[4] Inra ML, McCormick MG, Bagameri G, et al. Thoracic aortic dissection associated with involvement by small lymphocytic lymphoma/chronic lymphocytic leukemia: a possible underappreciated risk factor? Cardiovasc Pathol 2020; 45: 107179. 31865269 10.1016/j.carpath.2019.107179

[5] Sprague AH, Khalil RA. Inflammatory cytokines in vascular dysfunction and vascular disease. Biochem Pharmacol 2009; 78: 539-52. 19413999 10.1016/j.bcp.2009.04.029 PMC2730638

[6] Kamata S, Itou Y, Idoguchi K, et al. Abdominal aortic aneurysm with periaortic malignant lymphoma differentiated from aneurysmal rupture by clinical presentation and magnetic resonance imaging. J Vasc Surg Cases Innov Tech 2018; 4: 95-8. 29942890 10.1016/j.jvscit.2018.03.003 PMC6012996

[7] Pontailler M, Fabre D, Hocquemiller-Khalife T, et al. Thoraco-abdominal aortic aneurysm rupture secondary to lymphocytic lymphoma. Interact Cardiovasc Thorac Surg 2017; 24: 156-7. 27659154 10.1093/icvts/ivw310

[8] Kontopodis N, Galanakis N, Antoniou SA, et al. Meta-analysis and meta-regression analysis of outcomes of endovascular and open repair for ruptured abdominal aortic aneurysm. Eur J Vasc Endovasc Surg 2020; 59: 399-410. 31932143 10.1016/j.ejvs.2019.12.023

[9] Kumar R, Dattani N, Asaad O, et al. Meta-analysis of outcomes following aneurysm repair in patients with synchronous intra-abdominal abdominal malignancy. Eur J Vasc Endovasc Surg 2016; 52: 747-56. 27592036 10.1016/j.ejvs.2016.07.084

[10] Williamson AE, Annunziata G, Cone LA, et al. Endovascular repair of a ruptured abdominal aortic and iliac artery aneurysm with an acute iliocaval fistula secondary to lymphoma. Ann Vasc Surg 2002; 16: 145-9. 11972243 10.1007/s10016-001-0157-x

